# Abscission zone metabolism impacts pre- and post-harvest fruit quality: a very attaching story

**DOI:** 10.3389/fpls.2024.1524893

**Published:** 2025-01-27

**Authors:** Timothy J. Tranbarger, Francisco R. Tadeo

**Affiliations:** ^1^ UMR DIADE, IRD Centre de Montpellier, Institut de Recherche pour le Développement, Université de Montpellier, Montpellier, France; ^2^ Centro de Genómica, Instituto Valenciano de Investigaciones Agrarias (IVIA), Valencia, Spain

**Keywords:** abscission, ethylene, jasmonates, abscisic acid, fleshy fruit, ripening, reactive oxygen species (ROS), auxins

## Abstract

The function of abscission zones (AZs) determines the timing of fleshy fruit abscission, with important consequences not only for the optimal fruit harvest, but also on the overall final fruit quality. In this context, chemical treatments are commonly used at different stages of fruit development to control fruit abscission, which can also have positive or negative effects on fruit quality. In the current review, we examine commonly used chemicals that affect the metabolic activity in the AZs of fleshy fruit, in addition to their effects on fruit quality characteristics. The main hormone metabolism and signaling in the AZ include that of ethylene, auxin, abscisic acid and jasmonates, and the molecular components that are involved are covered and discussed, in addition to how these hormones work together to regulate AZ activity and hence, affect fruit quality. We focus on studies that have provided new insight into possible protein complexes that function in the AZ, including multiple MADS-box transcription factors, with potential overlapping regulatory roles which exist between AZ development, ethylene production, AZ activation, fruit ripening and overall fruit quality. The view of the AZ as a cross roads where multiple pathways and signals are integrated is discussed.

## 1 Introduction

**Figure 1:**
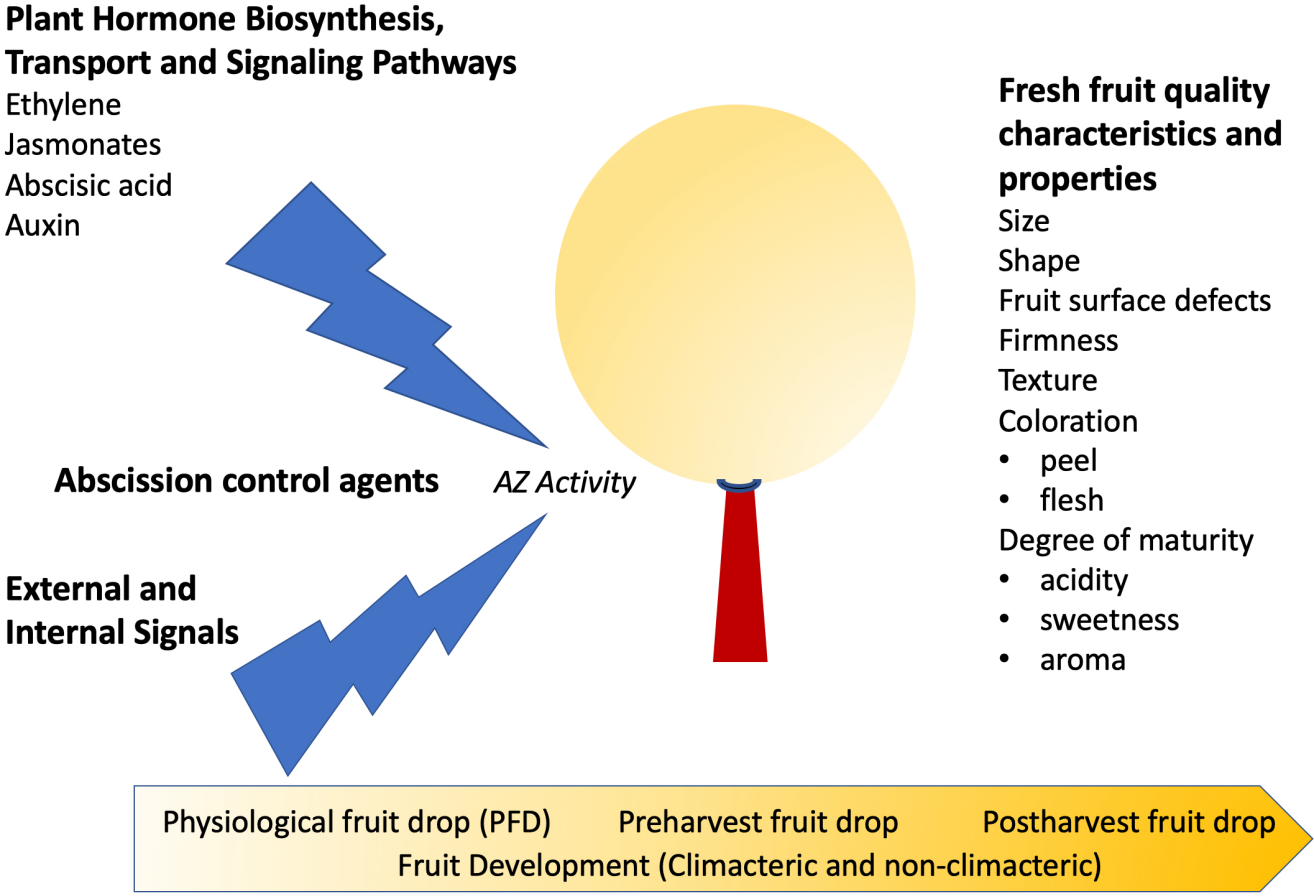
Plant hormones and abscission control agents that affect abscission zone (AZ) activity and fruit quality characteristics. Fleshy fruit can undergo abscission at different stages of fruit development, and it is still unclear whether the underlying mechanisms are the same.

Fresh fruit quality is very difficult to define because it must include the characteristics and properties that a fruit must attain to satisfy the requirements and needs of all the components of the production chain. Given the different and sometimes contrasted interests of producers, packers, distributors, sellers and consumers, it is comprehensible that the definition of fruit quality depends on the perspective. If we consider the expectations of the fruit consumers (i.e. the final component of the production chain) each variety has a characteristic fruit size and shape, and the changes that may occur within them, for environmental reasons or due to the cultural practices applied, may depreciate the market crop value and modify the consumers’ purchase decision. Other quality aspects of fruit include the presence of fruit surface defects, the coloration of the fruit peel and flesh, the firmness of the fruit, and the degree of maturity, which is determined mainly by the acidity and sweetness of the fleshy fruit tissues. These particular fresh fruit quality attributes are affected by both the activity of different physiological processes and the production practices carried out before harvest, in addition to the postharvest practices employed to extend shelf-life. In this context, the activity of the fruit abscission zone (AZ) plays a fundamental role to maintain and even enhance some of the fresh fruit quality attributes outlined above (**Figure 1**).

Abscission zones (AZs) are specialized tissues where the metabolic and structural modifications necessary to carry out organ detachment (abscission) from the main plant body are executed, and have essential functions in the detachment of flowers, developing and ripening fruit, all of which affect the overall harvest quantity and quality of fleshy fruits (for recent reviews, see Patharkar and Walker, 2018; Meir et al., 2019; Tranbarger and Tadeo, 2020). Cell separation in the AZs of flowers and fruits is triggered by both developmental and environmental cues. Many crop species flower profusely, and both unpollinated flowers and flowers that do not hold preferential positions in the canopy or inflorescence, naturally detach once the first fruits are initially set and begin to develop (Stephenson, 1981). In addition to flower drop, a considerable number of young developing fruits detach during the so-called “physiological fruit drop” (PFD). This self-regulatory mechanism for natural fruit abscission effectively adjusts fruit load to the plant’s nutritional status (Addicott, 1982). The outcome of the fruit set period, which determines the number of developing fruits per plant, has a significant impact on final fruit size. Thus, the higher the intensity of fruit abscission during the fruit set period, the larger the fruit size will be at full maturity. Likewise, fruits that continue to develop when fruit set is poor are much larger than the standard size for the variety. However, each variety is marketed with a standard and distinctive size so that when fruit set is poor, fruits show non-standard oversize. In addition to quality loss due to fruit oversizing, other fruit characteristics are usually also affected. In citrus, for example, the surface of oversized fruits is very rough, the peel is thickest, and the final color is poor, and hence the external appearance is less appealing to consumers. Not all fruit crop species produce fruit of marketable size through self-regulating mechanisms, and therefore fruit thinning practices are required to achieve optimal fruit size (Dennis, 2000). Manual thinning (i.e. the removal of fruit by hand) is one of the production practices used to reduce the fruit load of fruit trees. Another possibility is the use of chemical thinning agents such as plant growth regulators (**Table 1**) that cause fruit loosening by activating the fruit AZs (Bangerth, 2000). Chemical thinning has been and continues to be used successfully with several fruit crop species to increase fruit quality and crop value (Pescie and Strik, 2004; Han et al., 2019; Bound, 2021; Costa and Botton, 2022; Continella et al., 2022; Torres et al., 2024). Metamitron (3-methyl-4-amino-6-phenyl-1,2,4-triazin-5-one) is a systemic herbicide that is taken up by leaves and transiently inhibits photosystem II electron transport when it reaches chloroplasts (McArtney et al., 2012). Since the beginning of the 2000s, metamitron formulations have been marketed for use as a chemical fruit-thinning agent by limiting carbohydrate supply, which triggers abscission of flowers and young developing fruit. The application of metamitron to apple, pear or peach tree varieties produces a significant reduction in fruit set and increases the final fruit size, thus improving overall crop quality (Elsysy et al., 2020; Farias et al., 2020; Rosa et al., 2022). In addition to fruit size, AZ activation may also enhance other quality attributes. For example, the varieties of *Pyrus sinkiangensis* Yü grown in China’s Xinjiang region produce high quality fruits called fragrant pears because of their abundant floral aroma (Volk and Cornille, 2019). However, Xinjiang pear trees produce mature fruit with both deciduous and persistent calyxes. The quality of fruit with the calyxes detached is superior to those that have retained it, as they contain more juice, display a better-balanced sweetness/acidity ratio, have smaller cores, fewer stone cells, and maintain the characteristic egg shape (Kurban et al., 2012; Bo-Hui et al., 2015). Thus, studies of calyx abscission in this East Asian pear species target an increased production of high-quality fruit (Pei et al., 2016; Zhou et al., 2018; Gong et al., 2020).

**Table 1:** Abscission control agents and their effect on fruit abscission and ripening.

Agent	Pathway targeted	Abscission effect	Ripening effect	References^1^
2-Chloroethylphosphonic acid (ethephon)	Ethylene	POSITIVE	POSITIVE	Edgerton and Greenhalgh (1969); Alferez et al. (2006); Cao et al. (2024); Hale et al. (1970); Looney (1972); Rugini et al. (1982)
1-Aminocyclopropane-1-carboxylic acid (ACC)	Ethylene	POSITIVE	POSITIVE	Uzquiza et al. (2014); Merelo et al. (2017); Torres et al. (2024); Yang et al. (2017); Waseem et al. (2019); Griesser et al. (2020)
Aminoethoxyvinylglycine (AVG)	Ethylene biosynthesis	NEGATIVE	NEGATIVE	Gomez-Cadenas et al. (2000); Yuan and Carbaugh (2007); Dal Cin et al. (2008); Silverman et al. (2004); Kim et al. (2024); Nguyen et al. (2024)
1-Methylcyclopropene (1-MCP)	Ethylene signaling	NEGATIVE	NEGATIVE	Greene and Schupp (2004); Pozo et al. (2004); Yuan and Carbaugh (2007); Peng and Fu (2022); Cao et al. (2024); Kim et al. (2024)
5-Chloro-3-methyl-4-nitro-1H-pyrazole (CMNP)	Ethylene	POSITIVE	POSITIVE	Hartmond et al. (2000); Alferez et al. (2006); Alferez et al. (2013); Sharma et al. (2017)
Methyl jasmonate (MeJA)	Jasmonate	POSITIVE	POSITIVE/ NEGATIVE^2^	Uzquiza et al. (2013); Uzquiza et al. (2014); Fidelibus et al. (2022); Zapata et al. (2014); Lv et al. (2018); Wu et al. (2022)
Coronatine	Jasmonate	POSITIVE	?	Burns et al. (2003); Pozo et al. (2004)
2,4-Dichlorophenoxyacetic acid (2,4-D)	Auxin	NEGATIVE	NEGATIVE	Agustí et al. (2006); Peng et al. (2013); Dongariyal et al. (2024); Frenkel and Dyck (1973); Ferguson et al. (1984); Alhassan et al. (2022)
Naphthaleneacetic acid (NAA)	Auxin	POSITIVE/ NEGATIVE^2^	POSITIVE/ NEGATIVE^2^	Anthony and Coggins (2001); Li and Yuan (2008); Zhu et al. (2011); Masia et al. (1998); Clayton-Cuch et al. (2021); Böttcher et al. (2022)
Other synthetic auxins^3^	Auxin	NEGATIVE	NEGATIVE	Sarooshi (1982); Alhassan et al. (2020); Alhassan et al. (2022); Mostert et al. (2024)
Nordihydroguaiaretic acid (NDGA)	ABA biosynthesis	NEGATIVE	NEGATIVE	Zhu et al. (2022); Zhang et al. (2009b); Wang et al. (2016); Tomiyama et al. (2021)
Fluridone	ABA biosynthesis	NEGATIVE	NEGATIVE	Zacarias et al. (1995); Zhang et al. (2009a); Zhang et al. (2009b)
Paclobutrazol (PP333)	Gibberellin biosynthesis	POSITIVE	POSITIVE	Mauk et al. (1986); Richardson et al. (1986); Zacarias et al. (1995); Chen et al. (2020); Liu et al. (2023); Wu et al. (2023)
Gibberellic acid (GA3)	Gibberellin	NEGATIVE	NEGATIVE	Sarooshi (1982); Stutte and Gage (1990); Zacarias et al. (1995); Dagar et al. (2012); Kuhn et al. (2020); Luo et al. (2024)

^1^Only the selected publications reporting the role of these chemicals in abscission and ripening are given.

^2^The effect of Methyl jasmonate (MeJA) and Naphthaleneacetic acid (NAA) on abscission and ripening is dose-dependent.

^3^3,5,6-Trichloro-2-pyridyloxyacetic acid (3,5,6-TPA), 2-(4-Amino-3, 5-dichloro-6-fluoropyridin-2yl) oxyacetic acid (Fluroxypyr), S-Ethyl-4-chloro-O-tolylooxythioacetate (MCPA).

Once fruit set is complete, fruit continue to develop and ripen until they reach maturity. During this ripening period is when important fresh fruit quality attributes, such as peel and flesh color, and flesh acidity and sweetness, are achieved. However, in many fruit crop species, fruit attachment weakens during ripening and wind induced branch movements can result in fruit detachment before full maturity. This process, known as preharvest abscission or preharvest fruit drop, not only determines the optimum harvesting period, but also makes it impossible to market a portion of the fruit load because the external appearance, color, aroma, and flavor of the detached fruits deteriorate rapidly upon hitting the ground. For another example, ripe fruit abscission of oil palm (*Elaeis guinenesis*) affects the quality of the fruit, and the palm oil obtained from the mesocarp. When ripe fruit shed from the bunch and fall to the ground they can be bruised and injured, which activates an endogenous lipase that releases free fatty acids (FFA) from triglycerides stored in the fruit mesocarp (Desassis, 1957; Morcillo et al., 2013). Therefore, AZ activation not only has a negative effect on the yield (if shed fruit are not included in the harvest) and increase labor costs (due to the need to pick up shed fruit from the ground) but also the quality of the oil (Corley and Law, 2001; Sambanthamurthi, 2002; Corley and Tinker, 2016). The high endogenous lipase activity that releases FFA in the mesocarp of bruised ripe fruit has a major impact on the quality of the oil because an FFA content of 45% is thought to be unfit for human consumption, hence, low-lipase oil palm lines have been identified and studied to introgress the trait into main elite genotypes (Ebongue et al., 2008; Morcillo et al., 2013). Furthermore, the acidification and decreased quality of the oil due to the increase in FFA released by the lipase in detached fruit is another reason why ongoing studies on ripe fruit abscission target the identification of markers, and/or non-shedding phenotypes for the development of either elite non-shedding or delayed-shedding genotypes (Fooyontphanich et al., 2021; Morcillo et al., 2021). Another interesting example or pre-harvest fruit drop is with date palm (*Phoenix dactylifera* L.). When fruits are shed at the immature green stage (i.e. the Kimri stage) when, cell elongation is still occurring, the dropped fruit that are picked up off the ground are greatly appreciated and consumed by local people, and are more beneficial to human health compared to the fruit eaten at the riper stages (Othmani et al., 2020).

The response of the AZ cells to abscission signals is regulated by the balance between auxin and ethylene processes, including biosynthesis, transport and signal transduction of both hormones (extensive reviews on this subject can be found in: Taylor and Whitelaw, 2001; Estornell et al., 2013; Meir et al., 2015; Tucker and Kim, 2015; Tranbarger and Tadeo, 2020). Thus, treatments with ethylene biosynthesis inhibitors such as aminoethoxyvinylglycine (AVG), which blocks ACC synthase activity (Yu and Yang, 1979) and ethylene antagonists such as 1-methylcyclopropene (1-MCP), which competes with ethylene for binding to receptors preventing downstream ethylene signal transduction (Sisler and Serek, 1997; Watkins, 2006), delay preharvest abscission and also fruit ripening, thereby improve fruit quality (Greene and Schupp, 2004; Yuan and Carbaugh, 2007; Arseneault and Cline, 2016; Babalık, 2021; Elmenofy et al., 2021; Kaur et al., 2021; Lwin and Lee, 2021). Treatments with synthetic auxins such as naphthaleneacetic acid (NAA) also delay preharvest abscission but, at the same time, increase ethylene production in the fruit mesocarp, which accelerates ripening and softening, thus deteriorates fruit quality (Yuan and Carbaugh, 2007; Li and Yuan, 2008).

It is also important to note that, in certain cases, the activation of AZs has a beneficial effect on fruit quality. Manual destemming of clusters to produce stemless table grapes or mechanical harvesting for wine or raisins results in an open wound at the stem end of the berry, usually called a wet stem scar, due to tissue debris that remains adhered to the pedicel. The mechanical damage leads to juice leakage and leaves the berry mesocarp exposed to dehydration and invasion by pathogenic microorganisms, which reduces shelf-life and berry quality. The use of abscission agents, such as the ethylene releasing agent ethephon (2-chloroethylphosphonic acid), the immediate metabolic precursor of ethylene 1-aminocyclopropane-1-carboxylic acid (ACC), methyl jasmonate or coronatine (a phytotoxin that mimics jasmonic acid isoleucine), all stimulate the detachment of mature grape berries (Fidelibus et al., 2007; Uzquiza et al., 2013, 2014; Ferrara et al., 2016; Fidelibus et al., 2022). All these treatments promote the formation of a dry stem scar probably through transdifferentiation of the stem end cells of the berry into a protective layer that minimizes the loss of berry quality.

The quality and commercial value of fresh fruit can also be deteriorated by the abscission process that may occur during postharvest storage. This problem affects citrus fruits and other fruits that are marketed in bunches such as grapes, cherry tomatoes, bananas or the tropical fruit longkong. The external and internal ripening of citrus fruit is not necessarily synchronized, which implies that the quality characteristics of the fruit peel and flesh are independently regulated (Tadeo et al., 2008). A green peel color of fruit from early varieties of oranges and clementines, or fruit harvested early to avoid winter frosts, deters purchase by consumers, even if the internal quality is commercially acceptable. Degreening treatments with ethylene gas in controlled environmental chambers result in chlorophyll degradation and unmasking, and synthesis of carotenoids (Wardowsky et al., 2006; Porat, 2008), making the fruit that acquire an orange coloration more acceptable for marketing. However, degreening can have a negative impact on other fruit quality characteristics as it accelerates senescence, promotes calyx abscission, and shortens shelf life (Wardowsky et al., 2006; Baldwin et al., 2014). Treatments prior to degreening with synthetic auxins such as 2,4-dichlorophenoxyacetic acid (2,4-D), 3,5,6-Trichloro-2-pyridyloxyacetic acid (3,5,6-TPA), 2-(4-amino-3, 5-dichloro-6-fluoropyridin-2yl) oxyacetic acid (Fluroxypyr) and S-ethyl-4-chloro-O-tolylooxythioacetate (MCPA) reduce calyx abscission and improve, in some cases, fresh fruit quality by reducing fruit weight and firmness loss and calyx browning (Sdiri et al., 2013; Gill et al., 2015; Alhassan et al., 2020, 2022; Mostert et al., 2024). Longkong (*Lansium domesticum* Correa.) is a non-climacteric tropical fruit of the Meliaceae family that develops in inflorescences, with bunches containing 25-40 units, that is consumed fresh in Southeast Asia. Longkong fruit may detach from the bunch naturally both during growth and development and after harvest, while post-harvest treatment with NAA or 1-MCP delays abscission of commercially harvested ripe fruit (Taesakul et al., 2012). Grape berry abscission is a common problem during postharvest storage of table grapes (Lavee, 2017; Meena et al., 2017). Grape berry is a non-climacteric fruit and abscisic acid (ABA) appears to play a role in the onset of ripening and during postharvest storage (Sun et al., 2010). In addition, ABA promotes the activity of cell wall hydrolases, including cellulases and polygalacturonases, in the pedicel-peduncle boundary AZ (Zhang and Zhang, 2009) therefore, controlling ABA biosynthesis during postharvest storage could prevent berry abscission. Treatment of grape bunches with nordihydroguaiaretic acid (NDGA), an inhibitor of 9-cis-epoxycarotenoid dioxygenase (NCED), the key enzyme in ABA biosynthesis (Han et al., 2019), delays the decline in the berry detachment force and reduces berry abscission (Zhu et al., 2022). Tomatoes harvested in bunches have a fresh appearance that is highly appealing to consumers. However, unlike individual tomatoes, the shelf-life and quality of bunch tomatoes is linked to stem and calyx desiccation and fruit abscission. Treatment with minerals, ascorbic acid, salicylic acid or 1-MCP maintains the brightness of the calyx and fruit appearance, and delays bunch fruit drop (Beno-Moualem et al., 2004; Lichter et al., 2006; Aktas et al., 2012; Zhu et al., 2022). Bananas are perennial monocotyledon herbaceous plants with a pseudostem consisting of dozens of narrow leaves and a tuberous rhizome or underground corm, a true stem that produces suckers that allow the plant to grow vegetatively (Lobo and Rojas, 2020). Each pseudostem produces a single inflorescence with female flowers that give rise to fruits known as “fingers”. Up to 20 fingers can grow in bunches known as “hands” and there can be between 5 and 20 hands per inflorescence depending on the variety. Bananas are usually marketed by hands with the crown attached, but can undergo a physiological disorder known as “finger drop”, during which individual fingers may detach from the hands due to the weakening of the peel at the pedicel-pulp boundary region (Luyckx et al., 2016). The separation region between the pedicel and the fruit pulp does not contain a true AZ, although cell wall remodeling enzymes are upregulated during the process of finger detachment in a pedicel rupture area (MbéGuié-A-MbéGuié et al., 2009). Separated fingers maintain, in principle, a good eating quality but the wound resulting from the detachment process reduces both consumer appeal and hence, the market value. In addition, the wound also represents an entry route for pathogens, which results in a reduced postharvest shelf-life of the fruit. Treatment of hands with gibberellic acid (GA_3_) (Salazar and Serrano, 2013) or a combination of 1-MCP and modified atmosphere packaging maintains banana fruit quality and reduces the occurrence of finger drop (Li et al., 2023b). Finally, the identification of oil palm fruit bunches that are ready to be harvested is determined by the number of detached fruits found on the ground below the ripening fruit bunch (Lai et al., 2023). However, as discussed above, detached fruits can be injured and activate endogenous lipase in the mesocarp that increases FFA content, which leads to decreased extracted oil quality. Furthermore, bunches are typically stored for some time periods before oil is extracted, which can increase ripe fruit abscission and lipase activity. Hence, to minimize acidity, fruit bunches need to undergo post-harvest heat treatments to inactivate the endogenous lipase (Morcillo et al., 2013). However, fruit bunches also need to be harvested at the time of maximum oil content, so these conflicting requirements (i.e. maximum oil content versus limiting oil acidity) require a large and increasing costly labor force, posing major challenges for the industry (Lai et al., 2023). Oil palm provides examples in which harvest and post-harvest activation of the AZs have numerous effects on fruit quality, quantity and overall harvest cost.

## 2 Hormonal crosstalk occurs between the maturing fruit and the fruit AZ that regulates ripening-related abscission

Fleshy fruits are traditionally classified into two broadly defined categories, climacteric and non-climacteric, according to their respiratory profile and the way in which ethylene is produced during ripening (Seymour et al., 2013). Non-climacteric fruits such as citrus, grape, olive, sweet cherry, or litchi respire and produce ethylene at basal levels during ripening and the subsequent period of over-ripening, marked by fruit aging and decay. This class of fruits produce ethylene by the so-called autoinhibitory system-1 (McMurchie et al., 1972; Lelievre et al., 1997), by which ethylene synthesis is maintained at low levels through ethylene perception mechanisms (Barry and Giovannoni, 2007; Liu et al., 2015). In contrast to non-climacteric fruits, climacteric fruits such as tomato, apple, banana, mango, or peach are characterized by a burst of respiration that is accompanied by a dramatic increase in ethylene production at the onset of ripening through the shift from autoinhibitory system-1 to autocatalytic system-2 ethylene production (Osorio et al., 2012; Chen et al., 2018). Non-climacteric fruits are harvested at horticultural maturity, with a balanced acidity to sweetness ratio appropriate for each destination market. In contrast, climacteric fruits can be harvested at physiological maturity, given that ripening of climacteric fruits does not stop when they are detached from the tree and therefore ethylene continues to have an effect, which shortens post-harvest shelf life and fruit quality. Indeed, autocatalytic ethylene production in climacteric fruits shortens shelf life and causes detrimental effects on fruit quality during postharvest storage if appropriate treatments are not applied (Zhang et al., 2017; Fernández-Cancelo et al., 2024).

Dynamic changes in the amounts of phytohormones, mainly auxins, ethylene, and ABA, slow down the expansion of fleshy fruits and promote maturation and ripening. The maintenance of a high content of active natural auxins, such as indole-3-acetic acid (IAA), or exogenous application of IAA or synthetic auxins at the end of the growth period cause a delay in ripening in both climacteric and non-climacteric fruits (Camarero et al., 2023; Chen et al., 2023; Chirinos et al., 2023; Lv et al., 2023). The decrease in physiologically active IAA content, typically observed during fruit maturation and ripening, is due to a reduction in the expression of biosynthetic genes and an increase in the expression of genes involved in IAA conjugation to amino acids (Böttcher et al., 2011; Fortes et al., 2015; Feng et al., 2021; Fenn and Giovannoni, 2021; Huang et al., 2022). Ethylene plays a central role in the regulation of natural ripening of climacteric fruits, not only through the sharp ethylene increase at the beginning of the process, but also when ethylene is applied exogenously, it accelerates ripening and, vice versa, inhibitors of ethylene biosynthesis or perception stop ripening. The onset of climacteric ripening with the shift from autoinhibitory system-1 to autocatalytic system-2 ethylene production is caused by ethylene biosynthesis, signaling, and response gene expression changes (for recent reviews, see Brumos, 2021; Fenn and Giovannoni, 2021). Abscisic acid plays important roles in the ripening of non-climacteric fruits for recent reviews, see Bai et al., 2021; Gupta et al., 2022; Perotti et al., 2023). Similar to the case of ethylene, the application of ABA or inhibitors of its biosynthesis, such as fluridone or nordihydroguaiaretic acid (NDGA), accelerates or delays fruit ripening, respectively (Zhang et al., 2009a; Villalobos-González et al., 2016; Wang et al., 2016; Lama et al., 2020). Abscisic acid content increases at the onset of ripening of non-climacteric fruits and, depending on the species, its accumulation is continuous, as in citrus (Feng et al., 2021) and olive (Camarero et al., 2023), or decreases transiently during the last stages of ripening, as in grapevine (Pilati et al., 2017). The increase in ABA content during ripening is controlled by the balance in the expression of genes related to ABA biosynthesis and catabolism (Wheeler et al., 2009; Pilati et al., 2017). However, it is interesting to note that there is increasing evidence that shows ethylene and ABA are involved in the regulation of ripening in both climacteric and non-climacteric fruits. The involvement of ABA in the ripening of climacteric fruits is apparently related to its modulation of the ethylene biosynthetic pathway. Thus, ABA treatment accelerates tomato fruit ripening through the upregulation of the genes that encode ACC synthases and ACC oxidases, principle enzymes for ethylene biosynthesis (Zhang et al., 2009a), while overexpression of the ABA receptor SlPYL9 also accelerates tomato ripening through enhanced ABA accumulation and a significant increase in ethylene production (Kai et al., 2019). On the other hand, deletion of SlNCED1, a key regulatory enzyme of ABA biosynthesis, blocks the changes in tomato fruit texture that occur during ripening by downregulating the expression of genes associated with cell wall metabolism (Sun et al., 2012). Although non-climacteric fruits only produce basal levels of ethylene during ripening, an acceleration of ripening-associated processes can be induced by treatments with ethylene gas, ACC, the immediate water-soluble metabolic precursor of ethylene, or ethylene-releasing compounds, such as ethephon (Barry and Giovannoni, 2007; Chen et al., 2018), These observations suggest the involvement of ethylene perception and signaling in the regulation of ripening of non-climacteric fruits (Chen et al., 2018; Alferez et al., 2021; Dorta et al., 2023).

In addition to changes in color, aroma and flavor during ripening, fleshy fruits also undergo cell separation processes that lead to changes in fruit texture and detachment from the plant. Fruit softening is due to the progressive loss of flesh firmness through the action of a combination of hydrolases and cell wall remodeling proteins on the primary cell walls (Shi et al., 2023; Su et al., 2023). In addition, the rate of softening in climacteric and non-climacteric fruits is mainly due to the antagonistic action of auxin and ethylene or ABA (Li et al., 2021b, 2022; Wang and Seymour, 2022; Chen et al., 2023; Mattus-Araya et al., 2023; Qin et al., 2023). As in the case of fruit softening, hydrolases and cell wall remodeling proteins cause the disassembly of primary cell walls in fruit-AZs, resulting in the progressive weakening of adhesion between adjacent cells, and ultimately leading to fruit abscission. In fact, abscission of maturing fruit can be considered as one of the terminal events of the ripening program (Sexton, 2001). There is a broad range of internal and external stimuli mediated by phytohormones that elicit an abscission response (for review, see Taylor and Whitelaw, 2001). The generally accepted model suggests that a reduction in auxin efflux from the subtending organ to the AZ enables ethylene to trigger abscission (Basu et al., 2013; Chersicola et al., 2017; Botton and Ruperti, 2019; Dong et al., 2021). Abscisic acid has also been implicated in triggering fruit abscission by acting as a hormonal sensor of the abscising stimulus that modulates the level of ACC and ethylene emission (Guinn, 1982; Gomez-Cadenas et al., 2000; Eccher et al., 2013; Wilmowicz et al., 2016). All these three phytohormones, auxin, ethylene, and ABA, play an active role in the ripening of fleshy fruits and organ abscission and, therefore, hormonal crosstalk between maturing fruit and fruit AZs may be associated with the weakening of the attachment of maturing fruit to the plant.

Maturing fruits are mostly detached through the function of AZs located at the fruit-pedicel boundary region, at the calyx-AZ (Tranbarger and Tadeo, 2020), which is traversed by the vascular system that connects the fruit with other plant organs and is embedded in fruit tissues (for examples, see Rascio et al., 1985; Henderson and Osborne, 1990; Roongsattham et al., 2016; Merelo et al., 2017). This location of the calyx-AZ at the stem-end region of maturing fruits strongly suggests that a potential crosstalk between fruit and calyx-AZ tissues may influence abscission activation during ripening. As there is a decrease in IAA content in the maturing fruit, it is deduced that cell-to-cell polar auxin transport to the calyx-AZ would be greatly reduced, favoring the action of other abscission-activating hormones such as ethylene and ABA. Ethylene gas has high diffusion rates within cells as well as across lipid membranes and may reach the calyx-AZ to activate abscission. Transporters have been described for the immediate metabolic precursor of ethylene, ACC, which may also allow it to readily reach the calyx-AZ (Hirner et al., 2006; Choi et al., 2019). Members of transporters from different families including the ATP-binding cassette (ABC) G-family, nitrate transporter/peptide transporter (NPF) family and multidrug and toxic compound extrusion (MATE) family, have been shown to import or export ABA over short and long distances (Anfang and Shani, 2021; Zhang et al., 2023), implying that ABA accumulated in maturing fruits may also reach the calyx-AZ and participate in the activation of abscission. Ethylene, ACC and ABA modulate the expression of genes associated with hormone metabolism in a hormonal environment with lower amounts of IAA, enhancing the abscission response and inducing the expression of hydrolases and cell wall remodeling proteins in calyx-AZ cells to favor mature fruit detachment.

## 3 Hormonal metabolism and signaling in fruit abscission zones during maturing fleshy fruit abscission

Developing fruits undergo a final ripening process in which their growth in size substantially slows down and culminates with the acquisition of the attributes that will make them edible and desired by consumers. During ripening, fruits develop towards senescence and, gradually, the force with which they are attached to the calyx generally declines. The molecular signals triggering maturing fruit abscission are poorly understood and it is also unclear whether they are the same in all fruit crop species, although the importance in the abscission process of certain growth-regulating metabolites such as auxin, ethylene, ABA and JA is supported by many research reports.

### 3.1 Auxin-related gene expression in maturing fruit abscission zones

Auxins are involved in the transition from fruit growth-to-ripening and the initiation of ripening (for recent reviews, see Brumos, 2021; Li et al., 2021b). A decline in the content of IAA in fruits seems to be critical to slow down fruit growth and launch the ripening process. Application of auxin transport inhibitors such as 2,3,5-triiodobenzoic acid (TIBA) or N-1-napthylphthalamic acid (NPA) promote ripening in grape berries (Yakushiji et al., 2001; Serrano et al., 2023), while treatment with the synthetic auxin naphthaleneacetic acid (NAA) during pre-véraison delays it (Böttcher et al., 2011; Ziliotto et al., 2012; Dal Santo et al., 2020). The onset of ripening in both climacteric and non-climacteric fleshy fruit is also characterized by a decrease in fruit adhesion to the calyx and similar as during ripening, treatment with synthetic auxins delays abscission in citrus, apple, pear, or mango (Anthony and Coggins, 2001; Yuan and Carbaugh, 2007; Dal Cin et al., 2008; Hagemann et al., 2014; Murayama et al., 2015). Therefore, in general auxins play an inhibitory role in fleshy fruit ripening. However, there are exceptions to this general rule that give rise to fruit with diverse qualities. For example, late ripening fruits of some citrus or olive cultivars retain high auxin content (Camarero et al., 2023; Lv et al., 2023). Interestingly, a high concentration of IAA in the fruit of melting-flesh peach is required to induce the peach 1-aminocyclopropane-1-carboxylate synthase1 gene (*PpACS1*), that encodes a key ethylene biosynthesis enzyme, and the burst of system-2 ethylene production (Tatsuki et al., 2013). Furthermore, in stony hard peaches, there is no increase in IAA compared with the melting flesh peaches, which have a large increase in IAA and high system-2 ethylene production. These examples suggest that in some cases there are systems that function to uncouple the auxin-ethylene control of ripening.

The decline in auxin content in fruit AZs results in increased ethylene sensitivity and the initiation of abscission (Meir et al., 2015). Changes in IAA content in fruit AZs can be due to alterations in auxin transport from the subtending senescing fruit, which affect auxin metabolism and/or cellular transport and auxin signaling in that tissue. Olive fruit ripening and abscission of maturing olive fruit are modulated by auxin content. Both the content of IAA and fruit detachment force (FDF) in fruits of the Picual olive cultivar decreased from 164 days after anthesis while the content of IAA continued an upward pattern, and FDF remained high in the Arbequina olive cultivar resulting in a delay in fruit ripening and abscission (Parra-Lobato and Gomez-Jimenez, 2011; Camarero et al., 2023). Mature fruit abscission in Picual was associated with downregulation of several auxin synthesis-related genes encoding anthranilate synthase beta subunit 1 and flavin-containing monooxygenase YUCCA, and the upregulation of a gene for GRETCHEN HAGEN 3 (GH3) acyl acid amido synthetase (Gil-Amado and Gomez-Jimenez, 2013; Briegas et al., 2020). In addition, the genes for several AUXIN RESISTANT1 (AUX1) and LIKE AUX1 (LAX1) auxin influx carriers were also downregulated in the fruit AZ. These changes in auxin synthesis and transport genes in the olive fruit AZ were mostly associated with downregulation of several auxin response genes, including members of the Auxin/Indole-3-Acetic Acid (Aux/IAA) and auxin response factor (ARF) families (Gil-Amado and Gomez-Jimenez, 2013; Briegas et al., 2020). Similar transcriptomic changes to those occurring during natural abscission of maturing olive fruits have also been described in the abscission of Védrantais climacteric melons (Corbacho et al., 2013). Ethylene treatment of maturing citrus fruit accelerates abscission and its effect on the calyx AZ was also associated with changes in auxin signaling (Cheng et al., 2015; Merelo et al., 2017). In this regard, it is interesting to note that Ciclev10030696m and Ciclev10000693m, homologs respectively of AtARF2 and AtARF17 from Arabidopsis, were upregulated in the calyx AZ of ethylene-treated fruits (Merelo et al., 2017). The activity of ARF2 and ARF17 is related, respectively, to floral organ abscission (Ellis et al., 2005; Okushima et al., 2005) and anther dehiscence (Xu et al., 2019), two cell separation processes, strongly suggesting that citrus homologs of these genes might play a key role in abscission of maturing fruits of citrus. Abscission of maturing fruits in *Elaeis oleifera*, the oil palm from South and Central America, could also be related to auxin metabolism and signaling (Morcillo et al., 2021). An RNA-seq study in the AZ located between the pedicel and mesocarp of fruits from a non-shedding oil palm genotype showed decreased expression of several Arabidopsis homologs related to IAA homeostasis, including a PIN-LIKE 5 that regulates intracellular auxin homeostasis, an IAR3-like amido hydrolase that releases free IAA from inactive IAA-amino acid conjugates, and a PINOID-like protein involved in signal transduction and auxin response, which might be associated with non-shedding character of this variant. Likewise, in the AZ during ripe fruit abscission of the African oil palm (*E. guineensis*), dynamic changes in the expression of genes encoding proteins involved in auxin related processes including transport, conjugation and signaling, were found during both ethylene induced and natural abscission that occurs in the field (Fooyontphanich et al., 2021). Interestingly, alterations in auxin-related genes in ripe oil palm fruit AZs are similar to those that have been described in the pedicel AZ of tomato flowers following artificial auxin depletion (Meir et al., 2010, 2015). This opens the door to the possibility that, whatever the stage of fruit development, the triggering of abscission may be associated with similar alterations in auxin-related genes. The KNOTTED1-LIKE HOMEOBOX PROTEIN1 (KD1) gene is highly expressed in tomato flower and leaf AZs, regulates tomato flower pedicel abscission via alteration of the auxin gradient and response (Ma et al., 2015; Sundaresan et al., 2021). The KD1 transcription factor appears to alter various regulatory pathways, in particular a disruption of the auxin response gradient in the pedicel AZ, which activates abscission. Early stages of tomato flower and fruit abscission are associated with decreases in the expression of auxin efflux and influx carriers such as SlPIN1, SlPIN6, SlPIN9 and SlAUX/LAX2 (Shi et al., 2017; Dong et al., 2021). Recent work suggests changes in the distribution of auxin flow across the abscission zone are likely more important than the auxin response system for the regulation of abscission (Shi et al., 2017; Dong et al., 2021). Similarly, hydrogen sulfide (H_2_S) inhibits tomato pedicel abscission apparently through the downregulation of genes associated with cell wall modification (Liu et al., 2022a). The authors proposed that H_2_S reconstructs a basipetal auxin gradient along the pedicel, which may in turn decrease the AZ sensitivity to ethylene and result in the inhibition of pedicel abscission. The critical role of polar auxin transport in the control of tomato fruit abscission appears to be linked to the expression of the transcription factor BEL1-LIKE HOMEODOMAIN11 (SlBEL11) in the pedicel AZ. Knockdown of SlBEL11 by RNAi causes premature fruit drop from the breaker stage of fruit development and this effect was associated with a decrease in the level of quercetin, an auxin transport inhibitor, in the pedicel AZ (Dong et al., 2024). In fact, quercetin treatment suppressed fruit drop in *SlBEL11*-RNAi (RNA interference) plants. SlBEL11, through the regulatory role of SlMYB11 in flavonoid synthesis, seems to adjust the auxin flux coming from the fruit to maintain the auxin response gradient in the pedicel AZ and, thus, prevents premature fruit drop (Dong et al., 2024). The activation of ethylene biosynthesis in the fruit-AZ of litchi that occurs in response to auxin depletion was reported to be associated with the upregulation of the genes for two transcription factors, LcARF5 and LcEIL1, involved in auxin and ethylene signaling respectively (Ma et al., 2024). LcARF5 activated the gene expression for the peptide ligand LcIDL1 and its receptor LcHSL2, while LcEIL1 also activated the expression of *LcIDL1* thus launching the cell separation process in the fruit-AZ. Therefore, the physiological signal derived from auxin depletion in the fruit-AZ of litchi resulted in the upregulation of both auxin and ethylene signaling mediators, LcARF5 and LcEIL1, which triggered abscission through the activation of the LcIDL1-LcHSL2 regulatory module (Ma et al., 2024).

Some treatments that improve postharvest quality in fruit bunches also affect auxin metabolism and cellular transport. The application of gibberellic acid (GA_3_) is successfully used in the production of seedless table grapes that are highly appreciated by consumers. In addition to favoring stenospermocarpy, GA_3_ reduces the occurrence of the physiological disorder known as rachis browning, which is characterized by the browning of the berry skin and rachis (Lichter, 2016; Suehiro et al., 2019). However, GA_3_ treatment has the trade-off of promoting abscission during storage. This abscission-promoting effect of GA_3_ seems to be related to a strong reduction of IAA content in the berry AZ (Yu et al., 2024). GA_3_ treatment results in seed growth arrest leading to loss of auxin synthesizing capacity of seed traces, while developing seeds in non- GA_3_-treated berries show elevated levels of expression of auxin biosynthesis genes VvYUC2 and VvYUC4 and auxin transporter VvPIN4 and VvAUX1 in the berry AZ (Yu et al., 2024). GA_3_ treatment reduced gene expression of ethylene receptors VvETR1 and VvETR2, and VvEIN4 in the berry AZ, which increased ethylene sensitivity in the tissue and consequently increased both the activity of several cell wall hydrolases and fruit abscission.

### 3.2 Ethylene-related gene expression and metabolism in mature abscission zones

Ethylene, structurally the simplest plant hormone, has long been viewed as a key regulator of organ abscission, given that ethylene gas is often released from organs prior to abscission, and treatments with ethylene can induce organ abscission (Jackson and Osborne, 1970; Addicott, 1982) (for a recent review see Botton and Ruperti, 2019). In fact, ethylene was first discovered as the active agent that promoted leaf abscission from illuminating gas (Bakshi et al., 2015). As discussed above, treatments with ethylene biosynthesis inhibitors or ethylene antagonists inhibit both fruit abscission and ripening. In addition, the list of fruit species that abscise in response to exogenous ethylene treatments is very long, suggesting an almost universal role for ethylene in fruit abscission. A series of earlier studies on the biochemical and molecular basis of ethylene biosynthesis during peach fruit abscission first demonstrated the molecular basis of ethylene production and how ethylene can regulate gene expression in the AZ (Ruperti et al., 1998, 2001, 2002). The studies demonstrated how 1- aminocyclopropane-1-carboxylate oxidase (ACO) activity, an ethylene biosynthesis enzyme, and gene expression closely paralleled ethylene evolution in the peach fruit AZ. They determined that the increase in ethylene in the AZ prior to abscission is primarily through the increased activity of ACO. They went on to identify ethylene induced transcripts in the AZ, which shows the specificity of the response to ethylene in the AZ (Ruperti et al., 2002). In the following sections, we focus on recent discoveries about the role of ethylene and the metabolic and transcriptional consequences that occur in the AZ, from the most extensively studied fleshy fruit species that includes representatives from both climacteric and non-climacteric species.

Tomato is the most extensively studied model for climacteric fruit ripening and abscission; however, it remains to be seen whether it is representative for all fleshy fruits. In any case, it is an important baseline to see how ethylene regulates tomato AZ pedicel function. Furthermore, both unfertilized flowers and fully ripe fruit can be shed through the function of the AZ located within the pedicel, so with tomato, both types of abscission must be considered given there may be similar functions at different developmental stages (Iwai et al., 2013; Ito and Nakano, 2015; Tranbarger and Tadeo, 2020). Indeed, while both unfertilized flowers and fully ripe fruit can be shed through the function of the same AZ located within the pedicel, the majority of the studies use flower abscission as the model of study (Meir et al., 2010). Therefore, here we will focus on what is known about the events that occur in the developing pedicel AZ, in particular the effects on ethylene production, and how these events may have consequences on mechanisms that determine fruit quality.

The tomato ripening mutant *Never-ripe* (*Nr*) has a loss-of-function for the ethylene receptor SlETR3, and has delayed floral abscission, providing genetic evidence of the importance of ethylene perception for organ abscission (Tucker et al., 1984; Wilkinson et al., 1995). In addition, a splice variant of the key suppressor of ethylene response, CONSTITUTIVE TRIPLE RESPONSE1 (CTR1), is targeted for degradation by the microRNA *miR1917* in the tomato fruit pedicel AZ (Wang et al., 2018). Overexpression of *miR1917* resulted in higher expression of genes involved in ethylene signaling and biosynthesis, enhanced increased ethylene emission and early pedicel abscission. During premature abscission of tomato flowers and fruits triggered by low light stress, the tomato CLAVATA3-WUSCHEL (SlCLV3-SlWUS) signaling pathway modulates the auxin response gradient, which causes an increase in ethylene production in the AZ, and premature fruit abscission (Cheng et al., 2022). The tomato ERF family transcription factor ETHYLENE-RESPONSIVE FACTOR 52 (SlERF52) gene is specifically expressed in pedicel AZs, and *SlERF52* expression is suppressed in plants with impaired function of MACROCALYX (MC) and JOINTLESS (J), both of which are key regulators of pedicel AZ development (Nakano et al., 2014; Wang et al., 2021). Furthermore, *SlERF52* suppression results in a decrease in cellulase and polygalacturonase gene expression, in addition to genes that regulate meristematic activities in pedicel AZs, including the tomato WUSCHEL (LeWUS) homologue, GOBLET (GOB), and LATERAL SUPPRESSOR (Ls) (Nakano et al., 2014). The results suggest that SlERF52 plays a key role in AZ transcriptional regulation during both AZ development and the activation stage. Indeed, a recent study found that SlERF52 regulates the gene expression of an aquaporin tonoplast intrinsic protein SlTIP1;1 (Wang et al., 2021). When *SlTIP1;1* is knocked out, abscission is delayed, while when *SlTIP1;1* is overexpressed abscission is accelerated. They found that SlTIP1;1 mediates abscission via an increase in cytoplasmic H_2_O_2_ concentrations and osmotic water permeability, which enhances turgor pressure and the necessary break force for cell separation of AZ cells. With regard to ethylene, they demonstrated a positive loop in which cytoplasmic H_2_O_2_ activates ethylene production that then activates *SlERF52* expression.

MADS-box proteins are known to play important roles in tomato pedicel AZ development and fruit ripening (Ito and Nakano, 2015; Li et al., 2023a). MADS-box proteins are also known to function together in complexes, and these interactions provide possible links between AZ development, activation and fruit ripening and qualities. First characterized by the *j* mutant, which lacks a pedicel AZ, the MADS-box J was shown to be essential for pedicel AZ development (Mao et al., 2000). Later, it was found that J can function as a complex with other MADS-box proteins, including MC and SlMBP21 (JOINTLESS 2; J2), that regulates both AZ development and activation (Nakano et al., 2012; Liu et al., 2014; Roldan et al., 2017). While SlMBP21 is involved in pedicel-AZ differentiation in tomato, no clear role for SlBMP21 in the regulation of fruit ripening has yet been described (Liu et al., 2014; Roldan et al., 2017; Soyk et al., 2017). However, mutations in genes associated with pedicel-AZ formation show pleiotropic effects (Vrebalov et al., 2009; Nakano et al., 2012; Yuste-Lisbona et al., 2016), and one report of a possible role for SlBMP21 in ripening comes from the observation by Joubert (Joubert, 1965) that an additional J gene, jointless-2 with incomplete action, did not show the slow ripening characteristic of the J2 mutation. Therefore, SlMBP21/J2 most likely has a positive role in tomato fruit ripening. In addition, down-regulation of SEPALLATA (SEP) homologs of strawberry, banana and apple inhibit ripening and prolong fruit shelf life, pointing to a conserved positive role of SEP genes in controlling fruit ripening (Seymour et al., 2011; Ireland et al., 2013; Elitzur et al., 2016). Protein complexes formed by J, MC, and SlMBP21 confer the auxin/ethylene regulation that determines the activation of abscission and also regulate the meristematic maintenance genes LeWUS, Blind (Bl), GOB, and Ls and the development of the pedicel-AZ (Nakano et al., 2012; Liu et al., 2014).

**Figure 2:**
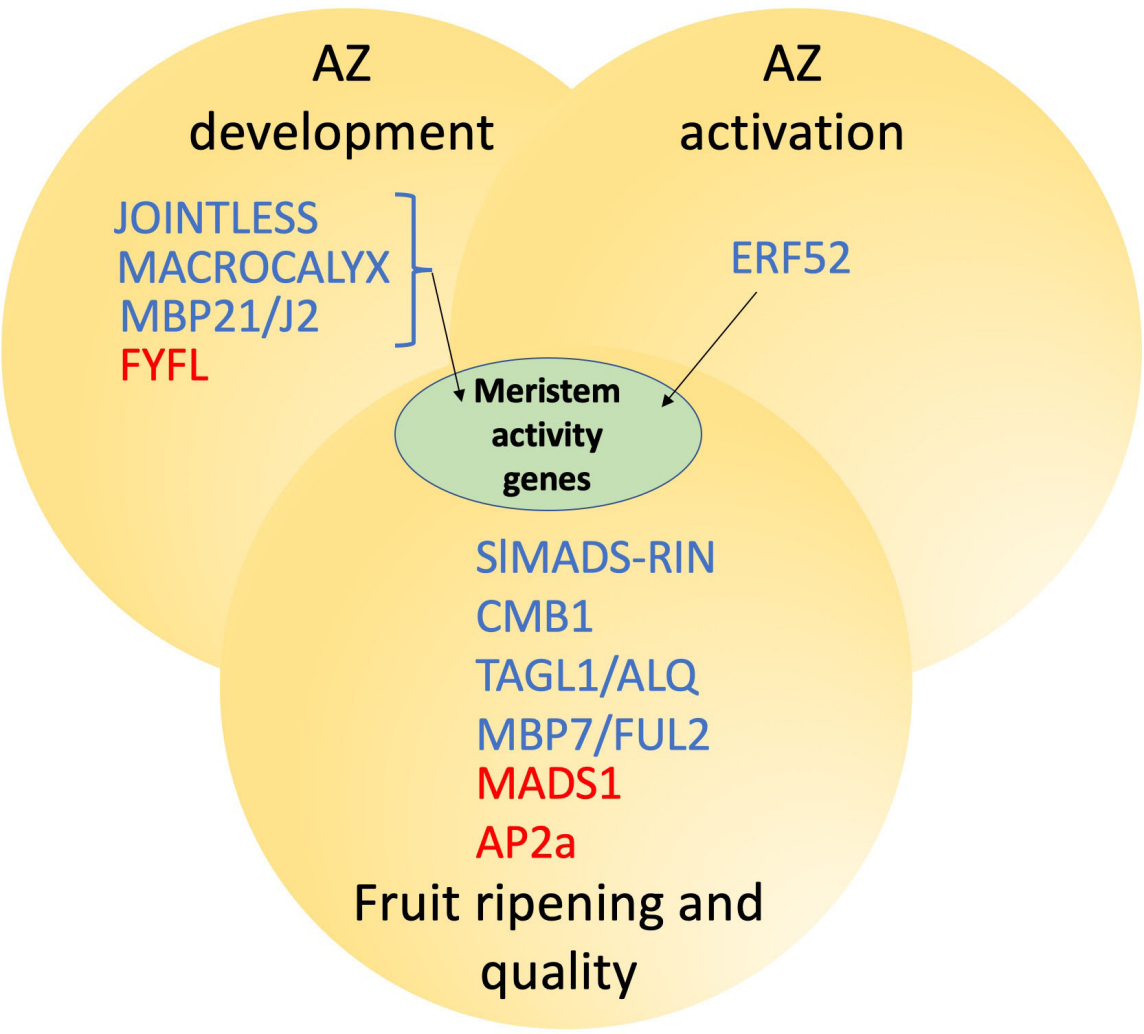
Protein interactions and transcriptional connections between tomato AZ function and fruit ripening. Proteins in blue are positive and those in red are negative regulators respectively. JOINTLESS, MACROCALYX and MBP21/J2 form a complex and can activate meristem activity genes.

Another tomato MADS-box protein gene, FOREVER YOUNG FLOWER-LIKE (SlFYFL), an ortholog of Arabidopsis FYF/AGL42, is highly expressed in the tomato fruit AZ (Xie et al., 2014). In tomato plants ectopically expressing *SlFYFL*, the development of the fruit AZ is impaired and ethylene production is reduced possibly due to the downregulation of ethylene biosynthesis pathway genes, along with several ethylene-response genes. Furthermore, carotenoid accumulation is also reduced in fruits of these 35S:FYFL plants but, in contrast, postharvest fruit storage is improved (Xie et al., 2014). In fact, SlFYFL was found to interact with other important fruit related MADS-box proteins including SlMADS-RIN, SlMADS1 and in particular with J, which is involved in AZ development. Similarly, the tomato homologs of MADS-box SEPALLATA 4 (AT2G03710), SlCMB1, SlMADS-RIN, SlMBP21 and SlMADS1, are all involved, in one way or another, in flower and fruit abscission and/or fruit ripening and also modify ethylene metabolism (Vrebalov et al., 2002; Dong et al., 2013; Liu et al., 2014; Xie et al., 2014; Li et al., 2017; Roldan et al., 2017; Zhang et al., 2018; Xing et al., 2022; Zhang et al., 2024a, b). Yeast two-hybrid assays revealed that SlMBP21 interacts with APETALA2a (SlAP2a), TOMATO AGAMOUS-LIKE1/ARLEQUIN (TAGL1/ALQ), J, MC, MADS-RIN and SlMADS1, while SlMADS1, in addition to SlMBP21, interact with MC and MADS-RIN (Xing et al., 2022; Zhang et al., 2024a, b). On the other hand, SlCMB1 also interacted in yeast two-hybrid assay with TAGL1/ALQ, SlMADS1 and MADS-RIN (Zhang et al., 2018). The TAGL1/ALQ, the tomato ortholog of the duplicated SHATTERPROOF (SHP) MADS box genes of Arabidopsis, is also necessary for fruit ripening (Vrebalov et al., 2009). The *Alq* mutation caused by ectopic overexpression of TAGL1 (Giménez et al., 2010) affects the reproductive development of tomato plants by producing defects in the formation of the style and pedicel AZs and homeotic alterations that affect the identity of floral organs by converting sepals into fruit-like organs (Pineda et al., 2010). TAGL1/ALQ transcriptional activity regulates ethylene production and some fruit ripening characteristics such as carotenoid production and fruit stiffness (Vrebalov et al., 2002; Itkin et al., 2009; Giménez et al., 2010). Therefore, TAGL1/ALQ has a positive role in tomato fruit ripening. The involvement of TAGL1/ALQ in tomato fruit ripening perhaps occurs through its role in the positive regulation of ethylene production (Jeon et al., 2024). TAGL1/ALQ’s role in the development of style- and pedicel-AZ is potentially through its relationship with J (Pineda et al., 2010). Finally, different tomato MADS-box proteins (TDR4/FUL1, MBP7/FUL2, TAGL1/ALQ, TAG1, TAG1, MBP21, and TDR5) have been identified as potential MADS-RIN interactors (Martel et al., 2011; Bemer et al., 2012). Amongst these MADS-box proteins, MBP7/FUL2, is involved in tomato style AZ formation and ripening, given that *MBP7/FUL2* overexpression blocks style abscission and extends tomato fruit shelf life, possible through functions in modulating post-harvest water loss (Wang et al., 2014). In addition to the participation of MBP7/FUL2 in style abscission and the regulation of tomato fruit shelf-life, MBP7/FUL2 is also involved in flower pedicel abscission (Cheng et al., 2022). Overall, these studies provide insight into possible protein complexes, which include many MADS-box transcription factors with potential overlapping regulatory roles which exist between AZ development, ethylene production, AZ activation, and fruit ripening and overall fruit quality (**Figure 2**; **Table 2**).

**Table 2:** Components with regulatory roles in the AZ related to metabolism and effects on fruit quality discussed in this review.

Gene name	Pathwaytargeted	Abscission effect	Ripening effect	References
KNOTTED1-LIKE HOMEOBOX PROTEIN1 (KD1)	Auxin transport	POSITIVE	?	Ma et al. (2015); Sundaresan et al. (2021)
HOMEOBOX15A (SlHB15A)	AZ activation Jasmonate metabolism	NEGATIVE	?	Liu et al. (2022b)
BEL1-LIKE HOMEODOMAIN11 (SlBEL11)	Auxin transport	NEGATIVE	?	Dong et al. (2024)
MicroRNA1917/CONSTITUTIVE TRIPLE RESPONSE1 (CTR1) Module	Ethylene biosynthesis and signaling	POSITIVE	POSITIVE	Wang et al. (2018)
CLAVATA3-WUSCHEL (SlCLV3-SlWUS) Module	Auxin response Ethylene biosynthsis	POSITIVE	?	Cheng et al. (2022)
ETHYLENE-RESPONSIVE FACTOR 52 (SlERF52)	AZ development and activation	POSITIVE	?	Nakano et al. (2014); Wang et al. (2021)
NEVER-RIPE (NR/SlETR3)	AZ activation Ethylene response	POSITIVE	POSITIVE	Tucker et al. (1984); Lanahan et al. (1994); Wilkinson et al. (1995)
MADS-box transcription factor FOREVER YOUNG FLOWER-LIKE (SlFYFL)	AZ activation Ethylene biosynthesis	NEGATIVE	NEGATIVE^2^	Xie et al. (2014)
MADS-box transcription factor JOINTLESS (J)	AZ development	POSITIVE	?	Mao et al. (2000)
MADS-box transcription factor SlMBP21/J2	AZ development Ethylene and auxin homeostasis	POSITIVE	POSITIVE	Joubert, 1965); Liu et al. (2014); Li et al. (2017); Roldan et al. (2017); Zhang et al. (2024a); Zhang et al. (2024b)
MADS-box transcription factor MACROCALYX (MC)	AZ development	POSITIVE	?	Nakano et al. (2012); Liu et al. (2014)
TOMATO AGAMOUS-LIKE1/ARLEQUIN (TAGL1/ALQ)	AZ development	POSITIVE	POSITIVE	Vrebalov et al. (2002); Itkin et al. (2009); Pineda et al. (2010)
MADS-box transcription factor FRUITFULL2 (MBP7/FUL2)	AZ activation Auxin gradient Ethylene response	POSITIVE	POSITIVE^3^	Bemer et al. (2012); Wang et al. (2014); Cheng et al. (2022)
^1^MADS-box transcription factor SlMADS-RIN	Fruit ripening pathways	?	POSITIVE	Xie et al. (2014)
^1^MADS-box transcription factor SlMADS1	Fruit ripening pathways	?	NEGATIVE	Dong et al. (2013); Xie et al. (2014)
^1^MADS-box transcription factor SlCMB1	Fruit ripening pathways	?	POSITIVE	Zhang et al. (2018); Xing et al. (2022)
^1^APETALA2a (SlAP2a)	Fruit ripening pathways	?	NEGATIVE	Chung et al. (2010)

^1^Roles during abscission are unknown, but interact with other AZ development related MADS-box transcription factors.

^2^When SlFYFL is overexpressed ectopically, there is a decrease in ethylene emission in the fruit, postharvest storage of fruit is improved.

^3^Affects ripening in an ethylene-independent manner (Bemer et al., 2012).

The tomato Hybrid Proline-rich Protein (THyPRP) gene is specifically expressed in the tomato flower AZs and when silenced, pedicel abscission is inhibited (Sundaresan et al., 2018). In *THyPRP* silenced plants, a decrease in expression for ethylene biosynthesis genes, including ACS, ACO, most likely results in reduced ethylene production in the AZ, which inhibits the acquisition of the competence of the AZ cells to respond to ethylene signaling. Overall, these studies show the central role of the ethylene biosynthesis and signal transduction pathways during tomato pedicel abscission, not only at the activation stage, but also earlier during the differentiation of the AZ, with apparent roles in regulating meristematic AZ gene regulators. This points out the powerful molecular tools that can be discovered in relation to AZ activation, and their potential for applications in the maintenance of overall fruit quality.

Table grapes, a non-climacteric fruit, are susceptible to abscission during post-harvest storage and transport, which has negative impacts on their commercial value (Zoffoli et al., 2009). Whereas grapes are non-climacteric, ethylene has been shown to play an important role in regulating AZ activity and hence, grape quality (Hedberg and Goodwin, 1980; El-Zeftawi, 1982; Ferrara et al., 2016). A recent article examined the effects of preharvest treatments with either nano-calcium (nano-Ca) and CaCl_2_ (Cl–Ca) (Zhu et al., 2024). The study found that nano-Ca significantly increased the calcium content in fruits, rachis, and the fruit AZ, while it inhibited ethylene production through a decrease in the expression of *VvACO1*. In addition, grapes pretreated with nano-calcium had a higher AZ pectin calcium content, decreased the activities of polygalacturonase (PG) and pectinesterase (PE), delayed pectin degradation, reduced weight loss percentage, decay percentage, malondialdehyde (MDA) content, and relative conductivity, and maintained a higher berry detachment force (BDF) and lower berry abscission percentage. Another problem is the common use of gibberellic acid-3 (GA_3_) as a pretreatment to increase size and marketability that also increases the post-harvest grape berry abscission rate (Nakamura and Hori, 1981; Casanova et al., 2009; Zoffoli et al., 2009; Meneses et al., 2020). A recent article revealed a lower expression of auxin biosynthesis genes, IAA content, and expression of ethylene receptor genes in GA3-treated berry clusters which results in a higher sensitivity to ethylene and abscission (Yu et al., 2024). Overall, it appears that ethylene biosynthesis and perception play regulatory roles during post-harvest grape berry abscission.

### 3.3 Jasmonate-related gene expression and metabolism in maturing fruit abscission zones

Jasmonates (JAs) are a family of phytohormones involved in plant adaptation to biotic and abiotic challenges, but also control different aspects of plant development and defense (Wasternack and Strnad, 2018; Ghorbel et al., 2021; Luo et al., 2023). Methyl jasmonate (MeJA), a volatile member of the JA family, has been shown in pre- or post-harvest treatments to enhance fruit quality in a number of fruit crops, including cherry, medlar fruit, kiwifruit, apricot, plum and apple (Saracoglu et al., 2017; Öztürk et al., 2019; Faizy et al., 2021; Öztürk and Yücedag, 2021; Öztürk et al., 2022; Wang et al., 2024). The fruit quality characters enhanced by MeJA include the retention of fruit flesh firmness, maintenance of bioactive compounds, slowing of color changes, reduction of respiration, delay of harvest, reduced weight loss, showing that MeJA is an effective tool to maintain a wide variety of fruit attributes. In addition, JAs enhance and play a functional role in Arabidopsis floral organ abscission, and JA treatments also increase the rate of fruit abscission in a number of crops, including citrus, tomato, grape and apple (Hartmond et al., 2000; Kender et al., 2001; Beno-Moualem et al., 2004; Kim et al., 2013; Uzquiza et al., 2014; Fidelibus et al., 2022; Wang et al., 2024). Interestingly, apples treated with MeJA have a decreased amount of early fruit drop, and enhanced fruit drop rate at the mature stage of development, suggesting the AZ response to JA is developmentally regulated (Wang et al., 2024). In a recent study with tomato, the class III homeodomain-leucine zipper transcription factor, HOMEOBOX15A (SlHB15A) was demonstrated to be a negative regulator of pedicel abscission using flower explants as a model (Liu et al., 2022b). The SlHB15A transcription factor negatively regulates abscission by decreasing JA-isoleucine (JA-Ile) amounts in the AZ through inhibiting the expression of the gene for JASMONATE-RESISTANT1 (SlJAR1) involved in JA-Ile biosynthesis. The JAR1 enzyme catalyzes the conjugation of isoleucine to JA, the final step in the formation of the bioactive JA molecule JA-Ile that binds to the JA receptor, CORONATINE INSENSITIVE 1 (COI1) (Ghorbel et al., 2021). The study also provides evidence that the SlJAR1-dependent JA-Ile-induced abscission is both dependent and independent of ethylene action. Finally, the authors also suggest that in the absence of ethylene, the JA-dependent pathway may play an important role in non-climacteric fruit abscission. Overall, while it remains to be determined whether the metabolism of JAs in the pedicel AZ of ripe fruit play an important role in the timing of fruit abscission, it is clear that JA metabolites have special properties that enhance quality attributes and can affect the abscission process of a number of fruit crops.

### 3.4 Abscisic-related gene expression and metabolism in maturing fruit abscission zones

Abscisic acid (ABA), with well-known roles in the regulation of plant growth and development, responses to abiotic and biotic stresses, and multiple physiological processes, has also been shown to play important roles in fruit ripening of both climacteric and non-climacteric fruit (Bai et al., 2021; Gupta et al., 2022; Perotti et al., 2023). As a generalization, ABA appears to be a major regulator of non-climacteric fruit ripening, while ABA and ethylene act together synergistically or through crosstalk to regulate climacteric fruit ripening. ABA can induce ethylene biosynthetic genes and result in a higher ethylene production, or in other cases, the ABA response depends on a functional ethylene signaling pathway (Zou et al., 2022). Nevertheless, the effects of ABA in both types of fruit, whether with or without an ethylene burst during ripening, appear to be similar and include cell wall loosening, color, changes in metabolites, including sugars, phenolics, flavonoids and antioxidants, and flavor related to the production of esters and volatiles (Gupta et al., 2022). Therefore, ABA has major role in determining important attributes for fruit quality.

ABA has long been considered a positive regulator of abscission since it was found to play a role in abscission of leaf explants (Abeles, 1967). Furthermore, its role as an accelerator of leaf explant abscission was associated with increased ethylene production (Abeles, 1967; Jackson and Osborne, 1972; Sagee et al., 1980). In addition to leaves, ABA also stimulated abscission of fleshy fruits. Rasmussen and co-workers (Rasmussen, 1974) observed that the uptake of ABA by the peduncle of mature citrus fruit explants accelerated abscission by causing a decrease in the fruit removal force (FRF). In addition, they also observed that the drop in FRF in fruit explants producing ethylene was greater when they were also treated with ABA, suggesting that ethylene and ABA are likely to have a combined effect on fruit abscission. A later study by Gómez-Cadenas and co-workers (Gomez-Cadenas et al., 2000) showed that citrus fruit abscission induced by carbohydrate starvation produced by defoliation was triggered by increases in the levels of ABA and 1-aminocyclopropane-1-carboxylic acid (ACC), the immediate metabolic precursor of ethylene. ABA treatment of fully defoliated citrus trees accelerated fruit abscission while treatment with fluridone, an indirect inhibitor of ABA synthesis (Bartels and Watson, 1978; Moore and Smith, 1984), delayed abscission suggesting that ABA appeared to act as a mediator between carbohydrate deficiency and ACC (Gomez-Cadenas et al., 2000). Many table grape varieties show berry abscission during postharvest storage (Rizzuti et al., 2015). The shedding of berries in the clusters was associated with senescence-induced ethylene as application of 1-methylcyclopropene (1-MCP), an ethylene antagonist, prevented rachis browning and blocked abscission (Wang et al., 2019). Table grapes undergo a rapid and transient increase in ABA accumulation in both rachis and berries, while ethylene emission increased continuously during postharvest storage (Zhu et al., 2022). Berry abscission was associated with a high decrease in FRF and increases in the activity of cell wall remodeling enzymes (pectin methylesterase, polygalacturonase and cellulase) in berry-AZs. Treatment with nordihydroguaiaretic acid (NDGA), an inhibitor of 9-cis-epoxycarotenoid dioxygenase (NCED), to grape clusters increased FRF and reduced berry abscission, cell wall remodeling enzyme activity in berry-AZs, and ABA content and ethylene emission in both rachis and berries (Zhu et al., 2022). This effect of NGDA on ethylene emission and ABA accumulation appeared to be caused by reduced expression of VvNCED1 and VvACO1, key enzymes in ABA and ethylene biosynthesis respectively. Therefore, ethylene-induced postharvest abscission in grapes could be favored by the transport of ABA from the senescing berry to the berry-AZ, thus affecting ethylene synthesis.

## 4 The AZ at the cross roads to integrate pathways and signals related to development, whole plant physiological status, environment, adaptation and defense

Signaling within the AZ has a pivotal role in determining the timing of fruit abscission. The current model of AZ function is based on four stages, including 1) AZ development, 2) AZ competence to react to stimulus, 3) activation of abscission and 4) scar formation. All these stages can have an effect on the final outcome of the fruit quality, whether indirect or direct. A picture is emerging in which these stages may not be independent, and may be interlinked through the formation of transcriptional complexes whose activity may not only provide connections between the different stages of abscission, but also the final characteristics of the fruit. Clearly, we know that auxin and ethylene play important roles during the acquisition of competence and activation stages, but how they interact at the molecular level is still unclear. In addition, we know less about the roles of other hormones, including ABA and JAs. It appears that in many cases ABA and JA act indirectly through ethylene by stimulating an increase in the transcripts related to ethylene biosynthesis, which results in more ethylene production. However, in other cases, it may be that ABA or JA have independent roles, and their stimulatory activities are synergistic with ethylene. The details of these mechanisms remain to be determined, and crosstalk between signaling pathways is currently a major focus of abscission research.

The current review has focused on the AZ metabolism during ripe fruit abscission that effects fruit quality, but most of our knowledge about mature fruit abscission comes from work with the tomato pedicel AZ during flower abscission. Are the mechanisms of flower abscission the same for mature fruit abscission, or are there different mechanisms dependent on the developmental stage? In addition, we have not discussed important abscission regulatory pathways that have been discovered from other organ abscission models, in particular Arabidopsis. One important mechanism, the INFLORESCENCE DEFICIENT IN ABSCISSION (IDA) peptide with receptor-like kinases HAESA (HAE)/HAE-LIKE2 (HAL2) pathway, was first discovered to function during Arabidopsis floral organ abscission (Stenvik et al., 2008). There is now evidence that the IDA/HAE/HAL2 pathway also functions during fruit abscission, including litchi fruitlet abscission, the tomato pedicel AZ, mango, citrus, and oil palm (Estornell et al., 2015; Stø et al., 2015; Tranbarger et al., 2019; Li et al., 2021a; Mesejo et al., 2021; Rai et al., 2021; Lu et al., 2023; Ma et al., 2024). In particular, the recent article on litchi showed the litchi AUXIN RESPONSE FACTOR 5 (LcARF5) activates litchi LcIDL1/LcHSL2 gene expression, while the ETHYLENE INSENSITIVE 3-like transcription factor LcEIL3 activates LcIDL1 gene expression (Ma et al., 2024). This provides a possible mechanistic explanation for the interplay between auxin and ethylene to initiate abscission.

A recent study on Arabidopsis floral abscission found the secretory manganese superoxide dismutase (SOD), MSD2, may play an integrative role between the IDA/HAE/HAL2 pathway, ethylene, nitric oxide (NO) and ABA signaling components (Lee et al., 2022). The evidence suggests that MSD2 and ROS signaling functions upstream of the IDA/HAE/HAL2 pathway. Furthermore, during litchi fruitlet abscission, the DOF (DNA binding with one finger) transcription factor LcDOF5.6 functions to repress ROS accumulation in fruitlet AZ and fruitlet abscission (Ma et al., 2023). When LcDOF5.6 is silenced, the gene expression for the ROS production enzyme LcRbohD increases, which results in higher ROS accumulation in the AZ and fruitlet abscission. From these recent studies, it appears that ROS signaling may play an integrative role during organ abscission.
